# Hearing loss after radiosurgery-blame it on Cochlear dose or the radiation tool!

**DOI:** 10.1186/s13014-019-1390-1

**Published:** 2019-10-30

**Authors:** Harsh Deora, Manjul Tripathi

**Affiliations:** 1 Department of Neurosurgery, National Institute of Mental Health and Neurosciences, Bangalore, India; 2 Department of Neurosurgery, Post Graduate Institute of Medical Education and Research, Chandigarh, 160012 India

**Keywords:** Vestibular schwannoma, Gamma knife, Stereotactic radiosurgery, Hearing loss

## Abstract

While sudden hearing loss after stereotactic radiosurgery has been demonstrated in some cases a recent article by Linge et al. and have demonstrated the need for further discussion on this topic. We attempt to delineate the fact that the type of dosing regimen or technology used will not affect the hearing or radio-graphical control outcomes and thus should not be a consideration while administering treatment. Also we discuss the role of location of the lesion and vascularity and potential new therapies for this unexpected outcome after radiosurgery.

Dear Editor,

We read with great interest the article by Linge et al. [1] regarding the loss of functional hearing in patients with vestibular schwannomas (VS) after fractionated stereotactic radiotherapy (FSRT) and Cyberknife stereotactic radiosurgery (SRS). They reported that after 1 year after treatment, 84% of the SRS patients and 71% of the FSRT patients had preservation of useful hearing. At 3 years, a useful hearing had been retained in 27% of the SRS patients and 50% of the FSRT patients. Linge et al. [1] noted that preservation of Gardener-Robertson hearing class I or II had not differed significantly between the two treatment groups. For larger tumors, including medium-size ones, SRS should be considered first-line therapy. Tuleasca et al. [2] state that acute and subacute complications after SRS for VS are independent of the used radiosurgery device. They also state that a vestibular dose of more than 8 Gy was responsible for the appearance of vestibular symptoms and that corticosteroid use in these cases almost always results in resolution of the symptoms.

VS comprise 8% of all primary brain neoplasms and 16% of all benign brain lesions and are inherently slow growing in nature thus allowing the great potential of treatment by radiosurgery. Perhaps no other intracranial pathology garnered such enthusiasm as VS after its remarkable track record of functional preservation and tumor growth control with gamma knife radiosurgery (GKRS). Regarding the radiosurgical technology used, there is no difference in the radiographic tumor control rate among the options available. Radiographic control ranged from 88.5–100% in LINAC-based series, and 71–100% in GK series [3]. With longer follow up, tumor control rates decrease regardless of the technology used. Only tumor size had an impact on radiographic control, with smaller tumors (< 3 cm) showing the highest tumor control rate at comparable time intervals, regardless of the technology.

Analogous to radiographic control, hearing preservation decreased with longer follow-up irrespective of the technology. Combs et al. [4] reported hearing preservation of 90% at 1 year, which subsequently decreased to 69% at 10 years using LINAC-based technology. GKRS based series such as those by Hasegawa et al. [5] reported a decrease in hearing preservation from 54% at 3 years to 34% at 8 years. Various series have reported hearing loss akin to presbycusis post-GKRS [3]. Also, hearing loss in sporadic and neurofibromatosis type 2 (NF2) cases needs to be differentiated, as sporadic cases are usually unilateral thus have better word recognition scores as compared to NF2 cases where the hearing loss in the functionally normal ear could be disastrous.

In addition to the often-discussed mechanism of cochlear spearing, other factors influence hearing preservation. A higher auditory function at baseline and young age can favorably contribute to higher rates of hearing preservation after SRS while an injury to the vasa nervosa of the cochlear nerve can secondarily cause radiation-induced tumor edema and lead to acute hearing loss. Hasegawa et al. [5] reported that in patients receiving < 4 Gy to the cochlea, hearing preservation at 3 years was 80 and 70% at 8 years (in contrast to 55 and 34%, respectively, with higher cochlear dose). Bashnagel et al. [6] reported a cochlear dose < 3 Gy to have favorable prognostic outcome on hearing preservation while Boari et al. [7] reported the highest hearing preservation in patients < 55 years of age with Gardner–Robertson (GR) Class 1 hearing prior to SRS, 93% compared to 71% in patients > 55 years of age, and 49% for the overall population, independent of GR class and age. Similarly, Franzin et al. [6] associated GR Class 1 hearing and age < 54 years old as favorable prognostic factors for hearing preservation. Thus, it is the basal turn of the cochlea, which needs protection. The final dose should not be more than 4 Gy to more than 10% of the cochlea [4–8]. The importance of the location of the tumor as cited by the author is controversial at best. Moffat et al. [7] reported acute SNHL secondary to the sudden rise in intracanalicular pressure in 28/139 patients of medically arising VS while Sauvaget et al. and others [8–10] reported the similar phenomenon in lateral arising tumors. The safest modality of the radiosurgery tool is a matter of heated debate among various treatment modalities. However, most long-term results are from GK series. A systematic comparison by Gaevert et al. [11] have shown that introduction of new LINAC based technologies (high definition multi-leaf collimation, intensity modulation) has reduced the gap between GKRS and LINAC based technologies, in terms of dose planning. The beauty of SRS lies in maintaining high conformity while minimizing dose spillage to the surrounding organs at risk. For this purpose, GKRS Perfexion and ICON systems comply with all the planning objectives. With the use of multiple non-isocentric-modulated beams (Cyberknife) or intensity-modulated beams instead of multiple isocenters (LGK-PFX) or dynamic arcs (NTx-DCA), a lower dose will be spread around the lesion. Finally, the use of inverse planning will accomplish the most homogeneous treatment plannings. For neurosurgeons, the Gamma Knife is a simple and effective tool. LINAC based SRS, on the other hand, requires several QA checks by radiation technologists and physicists. Dose plan is also more conformal with GK because the multi-isocenter plan can be generated and delivered. With most LINACs (except newer LINACs with high dose rate) dose delivery of multi-isocenter plans takes a very long time, which is not practical for the management. That is the reason most LINAC plans are 3–5 isocenter plans but it adversely affects the selectivity of the plan.

In this article for single-dose treatments, a dose of 12 Gy was prescribed at the 80% isodose surrounding the PTV. For fractionated treatments, a dose of 54 Gy was prescribed at the 100% isodose with the 95% isodose surrounding the PTV. Here, FSRT was delivered in 30 daily fractions of 1.8 Gy over a period of 6 weeks. Within a range of doses used in various series, a lower dose had little to no appreciable difference in progression-free survival, and generally high rates of progression-free survival were reported across a wide range of delivered doses [4–8]. Thus, level 3 evidence-based guideline by Germano et al. [3] states that a dose of ≤13 Gy achieves radiographic control while minimizing adverse effects hence should be used while planning SRS for VSs. While recent changes have allowed us to use a fractionated dose regimen and thus increase the total dose thus allowing time for normal cells to repair themselves between treatments, thereby reducing side effects to the surrounding tissues such as brainstem, cerebellum, cochlea. In the present paper, those who received 54 Gy were divided into 30 daily fractions of 1.8 Gy over a period of 6 weeks. Therefore, in terms of biological effects, the prescribed radiation is more than 90gy. Yet where possible we believe that the dosing should be according to the Noren’s policy, i.e., ‘the lowest irradiation doses that are therapeutically effective”. The author’s intention to give a higher dose in few cases is unexplained and needs to be understood in terms of his fractionation method used and if the same had any effect on the cochlear dose too.

In addition to the effect of steroids on the treatment of acute hearing loss, the role of Bevacizumab needs to be highlighted. Treatment with Bevacizumab results in a clinically relevant tumor-volume reduction and hearing improvement in some but not all patients treated with some possible treatment-related side effects. It is also useful to note that NF2 pathologic specimens stain for VEGF. Although the mechanism of action is unknown, it is presumed that bevacizumab inhibits VEGF-mediated angiogenesis within the tumor, resulting in its effect [3]. With our own experience with Bevacizumab resulting in hearing improvement on 2 cases, we understand that radiation induces an inflammatory reaction, characterized by an increase in the activated microglia, and cytokine release. This inflammatory reaction leads to a cycle of further cellular toxicity and tissue damage. A sudden increase in the intracanalicular component of VS may lead to a rise in intracanalicular pressure compromising the vascular supply of VII-VIII nerve complex. Bevacizumab is a humanized monoclonal antibody against VEGF and inhibits the blood-brain barrier (BBB) permeability and over-pruning of blood vessels. Bevacizumab has been reported to induce both tumor regression and hearing improvement in patients with NF2 associated VS.

## Data Availability

Not applicable.

## Abbreviations

FSRT: Fractionated stereotactic radiotherapy
GKRS: Gamma Knife Radiosurgery
LINAC: Linear accelarator
SRS: Stereotactic radiosurgery
VS: Vestibular schwanomma
